# Echocardiographic predictors of early in-hospital heart failure during first ST-elevation acute myocardial infarction: does myocardial performance index and left atrial volume improve diagnosis over conventional parameters of left ventricular function?

**DOI:** 10.1186/1476-7120-9-17

**Published:** 2011-06-03

**Authors:** Lilian P Souza, Orlando Campos, Clovis A Peres, Cristiano V Machado, Antonio C Carvalho

**Affiliations:** 1 Cardiology Department, Escola Paulista de Medicina, Federal University of Sao Paulo, UNIFESP, Brazil

**Keywords:** acute myocardial infarction, echocardiography, myocardial performance index, left atrial volume, ejection fraction

## Abstract

**Background:**

Left ventricular ejection fraction (LVEF) has been considered a major determinant of early outcome in acute myocardial infarction (AMI). Myocardial performance index (MPI) has been associated to early evolution in AMI in a heterogeneous population, including non ST-elevation or previous AMI. Left atrial volume has been related with late evolution after AMI. We evaluated the independent role of clinical and echocardiographic variables including LVEF, MPI and left atrial volume in predicting early in-hospital congestive heart failure (CHF) specifically in patients with a first isolated ST-elevation AMI.

**Methods:**

Echocardiography was performed within 30 hours of chest pain in 95 patients with a first ST-elevation AMI followed during the first week of hospitalization. Several clinical and echocardiographic variables were analyzed. CHF was defined as Killip class ≥ II. Multivariate regression analysis was used to select independent predictor of in-hospital CHF.

**Results:**

Early in-hospital CHF occurred in 29 (31%) of patients. LVEF ≤ 0.45 was the single independent and highly significant predictor of early CHF among other clinical and echocardiographic variables (odds ratio 17.0; [95% CI 4.1 - 70.8]; p < 0.0001). MPI alone could not predict CHF in first ST-elevation AMI patients. Left atrial volume was not associated with early CHF in such patients.

**Conclusion:**

For patients with first, isolated ST-elevation AMI, LVEF assessed by echocardiography still constitutes a strong and accurate independent predictor of early in-hospital CHF, superior to isolated MPI and left atrial volume in this particular subset of patients.

## Introduction

Early detection of patients with acute myocardial infarction (AMI) at risk of development of in-hospital congestive heart failure (CHF) is necessary to limit myocardial injury and left ventricular (LV) dysfunction. Non-invasive evaluation of LV function has been assessed by systolic as well diastolic echocardiographic indexes and related to short-term clinical outcome [1-8].

An increasing number of studies has reported the use of a combined index integrating systolic and diastolic LV function, the overall myocardial performance index (MPI)[9], for predicting short-term adverse outcome in AMI [10-14]. However, some included high-risk patients with multiple myocardial infarction [10,13] and previous history of CHF [13] or with non-ST elevation [10-13], while others studied only anteroseptal AMI [14], that could affect their results. Moreover, some authors [11,13,14] considered diverse in-hospital complications besides CHF (recurrent angina, reinfarction, death, arrhythmias, heart block, cardiac rupture and pericardial effusion), not always solely related to the extend of LV dysfunction in the acute phase of AMI. These factors may justify some controversy about the short-term independent prognostic significance of MPI in AMI patients, defended by some [10,11,14] but questioned by others [12,13]. Therefore, the value of MPI in predicting early in-hospital development CHF particularly in isolated, first ST-elevation AMI it is not yet fully established.

The left atrial (LA) volume measurement constitutes another new echocardiographic parameter that has been studied in post-AMI patients. Increased LA volume has been considered an independent predictor of adverse late outcome in patients with AMI and prior myocardial infarction and in patients with non-ST elevation AMI [15,16]. As far as we know, there is no description of the prognostic value of this index during the acute phase of first ST-elevation AMI.

We aimed to analyze the role of the MPI and LA volume compared to other conventional parameters of systolic and diastolic LV function in an homogeneous group of patients with a first, isolated ST-elevation AMI, in predicting early CHF during in-hospital evolution.

## Methods

### Patients

We studied prospectively 95 consecutive patients (58 ± 12 years old, 64 males) admitted to our coronary care unit with a first ST-elevation AMI, defined as characteristic chest pain lasting for more than 20 minutes, typical ST segment elevation > 1 mm in at least two contiguous leads associated with transient rise of creatine kinase MB. Exclusion criteria comprised: previous AMI, non-ST elevation AMI, early reinfarction, in-hospital death, previous coronary bypass surgery or angioplasty, left bundle branch block, non sinus rhythm, valvular heart disease, dilated cardiomyopathy and echocardiographic images of poor quality.

Patients were observed during daily in-hospital evolution, after receiving conventional clinical therapy (90% with betablockers and angiotensin converting enzyme inhibitors). Reperfusion therapy by thrombolysis or primary percutaneous angioplasty was instituted according standard guidelines [17]. Those without primary angioplasty were submitted to elective coronary angiography before hospital discharge for invasive risk stratification. In-hospital primary end-point was defined as the development of new-onset CHF in the first week of hospitalization, based on Killip functional status [18]. According to this classification, two groups of patients were established: without CHF (Killip class = I) and with CHF (Killip class ≥ II).

The study protocol was approved by the institutional ethical committee of our institution. Informed consent was obtained for each patient.

### Echocardiography

A comprehensive two-dimensional, spectral and color flow Doppler echocardiographic examination was performed in all patients within 24 hours of arrival at our coronary care unit (within 48 hours of chest pain). We used commercially available equipment (ATL-HDI 5000; Philips Medical System, Bothell, Wash.) with a P4-2 MHz transducer. Harmonic images and Doppler studies were recorded for further analysis. Indexes of global and segmental systolic function, diastolic function and combined systolic and diastolic functions were obtained. LV volumes and ejection fraction (EF) were determined using the modified biplane Simpson's method by orthogonal apical views (2 and 4-chambers), using mean values of 3 cardiac cycles [19]. Wall motion score index (WMSI) was calculated by using a 17-segment model proposed by the American Heart Association [19]. LA volume was obtained from the apical 4-chamber view at end systole by the method of discs [20], indexed for body surface area. Pulsed Doppler curves of blood flow were assessed by the apical 4-chamber view. Mitral diastolic inflow velocities were obtained at the tip of leaflets; LV outflow systolic flow curves were obtained just below the aortic valve closure plane. Mean values of peak velocities resulted from 5 consecutive cardiac cycles. The following variables were calculated: ratio of mitral E/A wave diastolic velocities; deceleration time (DT) of early LV diastolic filling; patterns of mitral diastolic filling (normal, abnormal relaxation, pseudonormal and restrictive filling). Myocardial performance index (MPI) was calculated by the method proposed by Tei [6,21], derived from its components: isovolumetric contraction time (IVCT), ejection time (ET) and isovolumetric relaxation time (IVRT), as previously described [10,11]. A single investigator performed the echocardiographic exams. One experienced observer, blinded to clinical data, made further interpretation.

### Statistical Analysis

Clinical and echocardiographic variables were expressed by mean values ± 1 standard deviation or proportion of patients with a determined characteristic. Comparison of continuous variables was made by a 2-sample "t" Student test or Mann-Withney rank sum test. Discrete variables were compared by Chi-square or Fisher's exact test. Linear regression was used to study the relationship between two continuous variables. A receiver operating characteristic (ROC) curve analysis was performed to determine the optimal cut-off point of some echocardiographic variables. We used univariate analysis followed by a complete model of multivariate logistic regression analysis to identify independent predictors of early CHF, including clinical and echocardiographic variables altogether, studied with interactions. A "p" value < 0.05 was considered to be significant.

## Results

### Clinical data

All patients presented a first, isolated ST-elevation AMI with wall motion abnormalities shown on echocardiography. Prevalence of anterior location (57%) on EKG was not significantly different from inferior infarction (43%; p > 0.05). Thrombolysis or primary angioplasty was performed in the majority of patients (80%). Significant coronary artery disease (≥ 70% of obstruction) was present in at least one major epicardial vessel in 93% of patients who underwent coronary angiography.

During the first week of hospitalization (mean: 5 days), 29 (31%) patients presented CHF (Killip class II:16; III: 5; IV: 8). Univariate comparison of clinical variables in both groups of patients are expressed in Table 1. Those with early CHF were significantly older, with a higher level of creatine kinase MB release. There was no difference between the groups regarding history of hypertension, diabetes, site of infarction, reperfusion therapy, use of betablockers, ACE inhibitors and multivessel disease.

**Table 1:** Clinical variables according to absence or presence of early CHF

	CHF absent (n = 66)	CHF present (n = 29)	p
Age (years)	54.9 ± 10.2	65.8 ± 14.2	< 0.001
Systemic Hypertension	32 (49%)	18 (62%)	0.22
Diabetes Mellitus	12 (18%)	5 (17%)	0.91
Site of infarction			
Anterior	35 (53%)	19 (66%)	0.26
Inferior	31 (47%)	10 (34%)	
Treatment			
PTI	26 (39%)	10 (35%)	
Trombolysis	30 (46%)	10 (34.5%)	
Conservative	10 (15%)	9 (31%)	0.19
CKMB peak (iu/l)	205 ± 138	346 ± 239	0.004
Angiography			
Non obstructive	5 (8%)	1 (3%)	
One vessel disease	34 (51%)	10 (35%)	
Multivessel disease	22 (33%)	15 (52%)	
Not done	5 (8%)	3 (10%)	0.31

Legend: data expressed by mean ± dp or absolute values and frequency (%); CHF: congestive heart failure; PTI: percutaneous transluminal intervention; CK: creatine kinase; angiography: non obstructive: obstruction absent or < 70% of luminal diameter, one vessel disease: obstruction ≥ 70% of luminal diameter in one or more epicardial coronary arteries, multivessel disease: obstruction ≥ 70% of luminal diameter in ≥ 2 epicardial coronary arteries.

### Echocardiographic data

The echocardiographic variables of the two groups of patients are shown in Table 2. The variables significantly related to the development of early CHF by univariate analysis were: lower LVEF (p < 0.001), higher values of WMSI (p < 0.001), higher values of MPI (p < 0.01) and its component ET (p < 0.05). There was a tendency of abnormal diastolic patterns to be related to early CHF (p: 0.06). There was a tendency of restrictive pattern (DT < 140 ms), present in only 8 patients, to be associated with early CHF (p:0.06). The mean values of DT were not significantly different in patients with or without CHF.

**Table 2:** Echocardiographic variables according to presence or absence of early CHF

Echocardiographic data	CHF absent (n = 66)	CHF present (n = 29)	p
LVEF	0.51 ± 0.07	0.44 ± 0.07	< 0.001
WMSI	1.57 ± 0.31	1.91 ± 0.35	< 0.001
LVESVI (mL/m^2^)	18.4 ± 7.6	20.8 ± 8.7	0.18
LVEDVI (mL/m^2^)	37.3 ± 12.2	37.0 ± 11.0	0.88
LAVI (mL/m^2^)	18.7 ± 4.8	20.6 ± 5.7	0.10
E/A ratio	1.02 ± 0.35	0.99 ± 0.50	0.76
DT (ms)	210 ± 61.9	212.7 ± 66.8	0.85
DT ≤ 140 ms	4 (6.1%)	4 (13.8%)	0.24
Diastolic patterns			
Normal	28 (42.4%)	5 (17.2%)	0.06
Abnormal relaxation	27 (40.9%)	14 (48.3%)	
Pseudonormal	7 (10.6%)	6 (20.7%)	
Restrictive	4 (6.1%)	4 (13.8%)	
MPI	0.57 ± 0.14	0.65 ± 0.16	0.01
ET (ms)	262.3 ± 22.8	247.5 ± 31.8	0.03
IVCT (ms)	46.5 ± 24.2	49.5 ± 27.3	0.60
IVRT (ms)	102.0 ± 26.6	108.4 ± 29.4	0.29

Legend: data expressed by mean ± dp or frequency (%); CHF: congestive heart failure; LVEF: left ventricular ejection fraction; WMSI: wall motion score index; LVESVI: left ventricular end systolic volume index; LVEDVI: left ventricular end diastolic volume index; LAVI: left atrial volume index; E/A:ratio of mitral E and A wave diastolic velocities; DT:deceleration time; MPI: myocardial performance index; ET: ejection time; IVCT: isovolumetric contraction time; IRVT: isovolumetric relaxation time; ms = miliseconds, ml/m^2 ^= mililiters per square meter

Left ventricular end diastolic and systolic volumes, as well as indexed LA volumes were similar in both groups. No differences between the groups were found concerning other variables (E/A ratio, DT, IVCT and IVRT).

### Multivariate analysis

Table 3 summarizes the selected cut-off values of echocardiographic continuous variables with higher statistical significance (LVEF, WMSI and MPI), with respective diagnostic indexes and area under the curves. A LVEF ≤ 0.45 had the best diagnostic performance. An analysis of interaction between variables with highest clinical relevance was made, involving age, CKMB levels, diastolic restrictive pattern (DT < 140), LVEF, WMSI and MPI.

**Table 3:** Cut-off values of echocardiographic continuous variables with statistical significance with respective diagnostic indexes and area under curves

Variable	Cut-off	S (%)	E (%)	PPV (%)	NPV (%)	Area under curves ROC
LVEF	0.45	87.9	62.1	84.0	69.2	0.77
WMSI	1.8	65.5	75.8	54.3	83.3	0.77
MPI	0.57	79.3	54.6	43.4	85.7	0.67

Legend: S: sensitivity; E: specificity; PPV: positive predictive value; NPV: negative predictive value; ROC: receiver operator characteristics; LVEF: left ventricular ejection fraction; WMSI: wall motion score index; MPI: myocardial performance index.

The final model selected the variables demonstrated in Tables 4 and 5. LVEF was the single independent variable significantly related to in-hospital CHF in this series of patients. Those with a first ST-elevation AMI and a LVEF ≤ 0.45 obtained by echocardiography at admission showed a higher and significant chance of developing CHF in the first week of hospitalization (odds ratio [OR] 17.0; 95% confidence interval [CI] 4.1 - 70.8; p < 0.0001). MPI alone failed to predict early CHF (p > 0.05) by logistic regression. Only when conditioned to older age ( > 60 years old), a MPI ≥ 0.57 was associated to in-hospital CHF (OR 13.7; 95% CI 2.7 - 68.6; p: 0.02), after interaction analysis. The LA volume and all other echocardiographic variables considered in logistic regression (WMSI, LV systolic volume, abnormal diastolic patterns, ET and IVCT), as well as the remaining clinical variables, were excluded by multivariate analysis.

**Table 4:** Logistic regression model with interaction analysis

Variable	Regression Coefficient	Standart Error	p
Constant	-3.1329	0.8195	0.1553
LVEF	2.8343	0.7275	0.0001
Age	-0.6230	1.0350	0.5472
MPI	-0.1780	0.8612	0.8362
MPI * Age	3.2431	1.3921	0.0198

Legend: LVEF: left ventricular ejection fraction; MPI: myocardial performance index, MPI*Age: interaction between variables MPI and age.

**Table 5:** Hazard ratio with confidence intervals of independent predictive variables

Variable	Hazard ratio	95% CI
LVEF ≤ 0.45	17.0	(4.1 - 70.8)
Age ≥ 60 and MPI < 0.57	0.5	(0.1 - 4.1)
Age ≥ 60 and MPI ≥ 0.57	13.7	(2.7 - 68.6)

Legend: CI: confidence interval; LVFE = left ventricular ejection fraction; MPI: myocardial performance index.

## Discussion

Our study re-emphasizes the power of LVEF, when analyzing other clinical and some more recent echocardiographic variables assessed at admission, in predicting early in-hospital CHF in the acute phase of an isolated, first ST-elevation AMI. Particularly in this subset of patients, an LVEF ≤ 0.45 was the strongest independent and highly significant variable (p < 0.0001) associated to the development of Killip class ≥ II. As a marker of myocardial dysfunction in the first week of hospitalization, it was superior to MPI or LA volume. These results were valid for both anterior and inferior location, irrespective of patient age.

Although univariate analysis showed MPI and WMSI to be significantly associated with early CHF, they were excluded in the multivariate analysis when all clinical and echocardiographic variables were included in the complete model of multiple logistic regression. Therefore, in first ST-elevation AMI patients, MPI alone was of limited short-term prognostic value in respect to in-hospital CHF. The interaction analysis between variables of our study demonstrated that only in patients older than 60 years or more, a MPI ≥ 0.57 could be predictive of early CHF in first ST-elevation AMI (p < 0.02).

In a retrospective study, Lavine [12] found similar results, describing the superiority of LVEF over MPI and diastolic patterns in predicting progressive development of CHF in the first 15 days of admission of a selected subset of patients with first ST-elevation AMI, without any clinical evidence of CHF at admission. According to this author, a LVEF < 0,40 was strongly related to CHF, and MPI alone had modest additional prognostic value. Schwammenthal *et al. *[13] also reported a LVEF ≤ 0.40 as a powerful and independent predictor of poor outcome one month after AMI. According to them, MPI had a low predictive accuracy with no additional early prognostic value in AMI.

On the contrary, other authors demonstrated the independent prognostic role of MPI in early stages of AMI, superior to LVEF [10,11,14]. Different clinical characteristics of their cohorts, like exclusive analysis of anteroseptal AMI [14], broader spectrum of clinical presentation including non-ST elevation AMI [10,11] and multiple infarcts [10] could in part explain their results. Non ST-elevation AMI constitutes a particularly heterogeneous subset of patients with different pathophysiologic, prognostic and therapeutic features than ST-elevation AMI patients [22], and are difficult to be compared when acute coronary syndromes are analyzed. Multiple infarcts are often associated with LV remodeling and greater systolic and diastolic dysfunction that could be expressed by higher MPI values, as it occurs in dilated cardiomyopathy [21]. MPI, as an integrated index of both systolic and diastolic LV function, could be previously elevated in patients with multiple large infarcts, even before the occurrence of additional myocardial injury induced by a novel episode of AMI. In patients with ST-elevation AMI and no previous infarction, without significant LV enlargement like the present series, MPI alone did not show any advantage over conventional LVEF in short-term prognosis.

We did not consider adverse events during in-hospital evolution that could be related to other factors and not to LV dysfunction, like recurrent angina or early malignant arrhythmias due to electrical instability that could lead to different results. In the series of Yuasa *et al *[14] among various other end-points (death, paroxysmal atrial fibrillation, ventricular tachycardia/fibrillation, advanced atrioventricular block, pericardial effusion and cardiac rupture), CHF was present in only 19% of the patients.

In our study, LVEF with a cut-off value of 0.45 as defined by a ROC curve presented has having the best diagnostic performance in predicting in-hospital CHF. Contrasting to the prognostic value of global assessment of LV systolic function by EF, our study did not demonstrate additional prognostic information of quantitative regional LV function analysis provided by WMSI. Because of interdependence of both variables (LVEF and WMSI) and some subjectivity when analyzing regional motion, WMSI could not have independent significance in predicting early CHF in these patients, as observed by others [11,14]. Like other authors [11,14] we did not find any independent prognostic value of conventional diastolic function parameters in the early echocardiogram of first ST- elevation AMI patients in predicting early CHF. A restrictive pattern in the exam at admission was present in only 8 (8.4%) patients in our series, and could be more prevalent in later periods of evolution [6], leading to a more significant prognostic information.

In our patients with first ST-elevation AMI, LA volume index was not significantly different between patients with or without CHF in the univariate analysis. This result was expected, since LA remodeling could not occur within 48 hours of initial presentation of a first AMI, because it is not a marker of acute changes in diastolic function and/or increased filling pressures, as the instantaneous transmitral diastolic flow parameters are [23]. Other studies described the long-term prognostic value of LA volume in AMI, including a significant proportion of patients with prior myocardial infarction, with a longer period of out-hospital follow-up [15,16]. It seems that for patients with isolated first ST-elevation AMI, echocardiographic structural indexes of LA volume are not useful for predicting early in-hospital CHF.

### Study limitations

Our results about the prognostic influence of abnormal diastolic pattern are not conclusive for first ST-elevation AMI, due to the limited number of patients with restrictive LV filling in the present series. We did not study the E/e' velocities ratio as an important marker of LV filling pressure, also useful in short-term prognosis after AMI [24], because e' waves derived from tissue Doppler tracings of septal and lateral mitral annulus were not available in all patients, limiting us to obtain this variable. Recent recommendations emphasize the use of averaged values of septal and lateral e' waves to calculate E/e' ratio in patients with segmental myocardial dysfunction, as it occurs in AMI [25]. So, this important surrogate of left atrial pressure can not be ruled out as a valuable prognostic index of early CHF in this subset of patients with a first ST-elevation AMI. Not only diastolic (E/e'), but also systolic (s') tissue Doppler-derived indices seems to be useful in early evaluation of patients with AMI and LV dysfunction [26], as well as new markers of myocardial deformation [27]. The prognostic role of these variables should be addressed in further studies in this clinical setting.

## Conclusion

In this series of patients with a first, isolated ST-elevation AMI without significant LV dilation, LVEF was the single independent predictor or early CHF. MPI alone or LA volume failed to predict in-hospital CHF during the first week of evolution in this particular subset of patients.

## Abbreviations

AMI: acute myocardial infarction; LVEF: left ventricular ejection fraction; MPI: myocardial performance index; CHF: congestive heart failure; LA: left atrial; WMSI: wall motion score index; DT: deceleration time; IVRT: isovolumic relaxation time; IVCT: isovolumic contraction time; ET: ejection time

## Competing interests

The authors declare that they have no competing interests.

## Authors' contributions

LPS: participated in the study design, carried out the echocardiographic examinations and draft the manuscript; OC: conceived the study, participated in its design, performed its coordination and contributed to write the manuscript; CAP: participated in the study design and performed the statistical analysis; CVM: contributed in the study design and reviewed the manuscript; ACC: participated in the study design and in the revision of the manuscript. All authors approved the final version of the manuscript.
